# *De Novo* Whole-Genome Sequencing of Two Pathogenic Pasteurella multocida Type D:6 Strains Isolated from Pigs

**DOI:** 10.1128/mra.00098-23

**Published:** 2023-04-03

**Authors:** Morag Livingstone, Pernille Jorgensen, Margaret McCall, Jill Thomson, David Longbottom

**Affiliations:** a Moredun Research Institute, Edinburgh, Midlothian, United Kingdom; b Scotland's Rural College, Aberdeen Veterinary Disease Surveillance Centre, Bucksburn, Aberdeen, United Kingdom; c Scotland's Rural College Veterinary Services, Veterinary and Analytical Laboratory, Edinburgh, Midlothian, United Kingdom; University of Maryland School of Medicine

## Abstract

Here, we report the complete genome sequences of Pasteurella multocida strains P504190 and P504188/1, which were isolated from the diseased lungs of a sow and her piglet, respectively. Despite the unusual clinical presentation, whole-genome sequence typing revealed both strains to be capsular type D and lipopolysaccharide (LPS) group 6, commonly found in pigs.

## ANNOUNCEMENT

Pasteurella multocida is a facultative anaerobic Gram-negative bacterial pathogen with the ability to infect a broad host range. P. multocida is responsible for significant economic losses in pigs worldwide, causing porcine atrophic rhinitis and respiratory tract disease, with the majority of isolates recovered from clinical cases being characterized as capsular type A, D, or F (1–3).

We report here the complete genome sequences of P. multocida strains P504190 and P504188/1, which were isolated from the lungs of a sow and her stillborn piglet, respectively, both of which exhibited unusual gross lesions consistent with a severe acute necrotizing bacterial-type pneumonia and pleurisy. Isolated strains were cultured on blood agar base number 2 with sheep blood (Thermo Fisher Scientific), and P. multocida was identified from colonies by matrix-assisted laser desorption ionization–time of flight mass spectrometry. Single colonies were grown aerobically at 37°C for 3 h in Oxoid nutrient broth (Thermo Fisher Scientific). Genomic DNA (gDNA) was extracted by lysis of bacterial pellets at 37°C in Tris-EDTA buffer containing lysozyme and RNase A and then was incubated at 65°C following the addition of proteinase K and SDS. DNA was purified using SPRI beads (Qiagen) and quantified with the Quant-iT double-stranded DNA high-sensitivity kit (Thermo Fisher Scientific). For Illumina sequencing, gDNA libraries were prepared using the Nextera XT library preparation kit and sequenced using the Illumina NovaSeq platform using a 250-bp paired-end protocol. For Oxford Nanopore Technologies sequencing, gDNA libraries were prepared using the rapid barcoding (SQK-RBK004) kit, loaded in a FLO-MIN106 (R.9.4.1) flow cell, and sequenced using a GridION system, with live base calling (Guppy v4.2.2). Illumina reads were adapter trimmed (Trimmomatic v0.30 with a sliding window quality cutoff value of Q15 [4]), and trimmed raw data analysis was performed on the Galaxy platform (https://usegalaxy.eu/) (5). Read quality was assessed using FastQC v0.73+galaxy0 (6) and NanoPlot v1.36.2+galaxy1 (7). Illumina sequencing resulted in 458,547 paired-end reads (average read length, 672 bp) and 800,353 paired-end reads (average read length, 554 bp) for P504190 and P504188/1, respectively. Nanopore sequencing produced 19,992 reads (average read length, 7,246.7 bp; read *N*_50_, 17,532 bp) for P504190 and 31,718 reads (average read length, 8,573.0 bp; read *N*_50_, 19,471 bp) for P504188/1. *De novo* assembly of Nanopore raw reads was performed using Flye v2.9+galaxy0 (8), followed by polishing with Illumina reads using Pilon v1.20.1 (9) after mapping with BWA-MEM v0.7.17.2 (10). Assembly produced single circular contigs (oriented to gene *dnaA*) of 2,327,408 bp (GC content, 40.31%) and 2,327,355 bp (GC content, 40.31%) for P504190 and P504188/1, respectively. The average genome coverage for P504190 Illumina and Nanopore read sequences was 94× and 60×, respectively, and that for P504188/1 sequences was 113× and 167×. Assembly metrics were calculated using QUAST v5.0.2+galaxy1 (11). The complete genomes were annotated using the NCBI Prokaryotic Genome Annotation Pipeline v5.0 (12). Default settings were used throughout for all utilized software packages.

The whole-genome sequences were queried in the Pasteurella multocida Bacterial Isolate Genome Sequence Database (https://ivsmlst.sund.ku.dk), which typed both strains as capsular type D and lipopolysaccharide (LPS) group 6 (13).

### Data availability.

The genome sequences are available in GenBank/EMBL/DDBJ under accession numbers CP110621 (P504190) and CP110620 (P504188/1). Raw sequence reads are available under BioProject accession number PRJNA896625.
